# Economical production of vitamin K_2_ using crude glycerol from the by-product of biodiesel

**DOI:** 10.1038/s41598-020-62737-x

**Published:** 2020-04-06

**Authors:** Chao Zhang, Daoji Wu, Huixue Ren

**Affiliations:** 1 School of Municipal and Environmental Engineering, Shandong Jianzhu University, JiNan, 250101 China; 2 Co-Innovation Center of Green Building, JiNan, 250101 China

**Keywords:** Environmental biotechnology, Industrial microbiology

## Abstract

Industrial waste, such as crude glycerol, was used for vitamin K_2_ by *B. subtilis* Z-15. Crude glycerol could be used instead of pure glycerin for vitamin K_2_ production. The combination of soybean peptone and yeast extract was more conducive to the synthesis of vitamin K_2_. The optimal composition of medium was obtained by response surface methodology. The results indicated that the optimal medium was as follows: 6.3% crude glycerol, 3.0% soybean peptone concentration and 5.1 g/L yeast extract. Under the optimal culture medium, vitamin K_2_ production was increased to 45.11 ± 0.62 mg/L. The fermentor test further proved that the use of crude glycerol affected neither the synthesis of vitamin K_2_ nor the growth of *B. subtilis*. These investigations could lay a foundation for reducing the pollution of crude glycerol, exploring a late model for vitamin K_2_ cleaner production.

## Introduction

Vitamin K_2_ refers to a series of naphthoquinone derivatives, which have a variety of physiological and pharmacological functions for the human body, and it is also called menaquinone-n (MK-n, n = 1–14), where n denotes the number of isoprene units in the side chain^1,2^. Studies have shown that vitamin K_2_ has effects on human coagulation, skeletal metabolism and cardiovascular disease treatment^3^. In addition, it can inhibit the proliferation of cancer cells^4^. Recent studies have found that vitamin K_2_, as an electronic carrier, also can be used to treat mitochondrial pathologies such as Parkinson’s disease and lateral muscle atrophy^5^.

Presently, the preparation methods of vitamin K_2_ typically use chemical synthesis and microbial fermentation, but natural K_2_ can only be obtained by microbial fermentation^6^. *Bacillus subtilis* has become the most important microorganism for vitamin K_2_ fermentation, due of its fast growth, easy cultivation, and high vitamin K_2_ content^7^. It is one of the ideal strains for industrial production of vitamin K_2_. For example, Mahdinia *et al*.^8^ reported that with a composition of 48.2 g/L of glycerol, 8.1 g/L of yeast extracts, and 13.6 g/L of soytone, vitamin K_2_ could reach 14.7 mg/L in *B. subtilis*. Hu *et al*.^9^ reported that a vitamin K_2_ yield of 31.18 mg/L in *Bacillus natto* was achieved under optimal conditions containing 53.6 g/L glycerol and 100 g/L soy peptone. It can be seen from the above studies that glycerol is used as a carbon source in many vitamin K_2_ studies. Although many researchers have studied production by microbial fermentation, the fermentation technology is still immature^10,11^. Moreover, the high cost of vitamin K_2_ production is one of the bottlenecks of industrialisation, which is directly related to the cost of the medium. However, few studies have been reported on the use of waste to produce vitamin K_2_. For example, soybean extract is a by-product of natto processing, so it is also cheap and an ideal nitrogen source. Sato *et al*.^12^ have shown that *B. subtilis* D200-41 could produce 60 mg//L vitamin K_2_ in a medium containing 10% soybean extract, 5% glycerol, 0.5% yeast extract, and 0.05% K_2_HPO_4_. From the above studies, it can be seen that glycerol is used as a carbon source in most of the studies, and glycerol is used in a very large amount, but no researchers have considered reducing the cost of the medium as far as glycerol is concerned.

Crude glycerol is a by-product of alcoholic fermentation and saponification of oils and fats. During the preparation of biodiesel, roughly 1 ton of crude glycerol is produced for every 9 tons of biodiesel produced^13,14^. With the increasing scale of biodiesel, the yield of crude glycerol will increase accordingly. Crude glycerol contains impurities—such as salt, methanol, and soap—in the preparation process^15^. It can be used in medicine, cosmetics, and food only after further purification and refinement^16^. However, the high cost of crude glycerol purification and refining is not economically viable, resulting in a sharp drop in the price of crude glycerol, which has essentially become industrial waste. If not treated in time, it may become a new pollution source^17^, thereby increasing the processing cost of biodiesel production enterprises and reducing economic benefits. The use of biotechnology to convert crude glycerol as a substrate into high-cost products has attracted increasing attention among researchers^18^. At present, the effective ways to utilise crude glycerol include: fermentation to produce 1,3-propanediol, succinic acid, etc.^19–22^. However, there is no research on the conversion of crude glycerol to vitamin K_2_ by microorganisms. The utilisation of crude glycerol provides a carbon source for vitamin K_2_ production, which reduces the cost of the medium. In addition, this process can offset the disposal costs of crude glycerol.

The principle of response surface method (RSM) is to fit the functional relationship between factors and response values through reasonable experimental design, and to find the optimal process through regression equation. Although some scholars used RSM to optimise the medium to improve the yield of vitamin K_2_, there was no relevant report in the use of RSM to optimise the crude glycerol medium to produce vitamin K_2_^2^. In this work, the goal was to investigate the feasibility of producing vitamin K_2_ from crude glycerol and to optimise the fermentation medium of *B. subtilis* Z-15 using RSM.

## Results

### Effect of different carbon sources on vitamin K_2_ production

Carbon source is one of the main constituents of the medium. Its main function is to provide the necessary energy for the life activities of bacteria and to construct the cell components of bacteria. At the same time, it also has certain effects on the metabolites of bacteria^23^. In this study, glycerol, glucose, fructose, sucrose, galactose, lactose, mannose, and sorbitol were used as carbon sources in the medium with a concentration of 5%. The effects of different carbon sources on vitamin K_2_ production were studied after shaking flask fermentation for 4 days. Results are outlined in Table 1.

**Table 1:** Effect of various carbon source on vitamin K_2_ production.

Carbon source	Concentration (%)	K_2_ yield(mg/L)	Biomass (g/L)	Prize(kg/dollar)
Glycerol	5	35.25 ± 0.76	2.84 ± 0.12	1.1–1.3
Sucrose	5	21.13 ± 0.52	3.44 ± 0.16	0.9–1.0
Glucose	5	22.20 ± 0.57	3.24 ± 0.10	0.60–0.65
Fructose	5	23.01 ± 0.46	3.06 ± 0.14	1.5–1.6
Lactose	5	19.16 ± 0.43	2.30 ± 0.16	2.0–2.1
Galactose	5	19.21 ± 0.52	2.34 ± 0.16	5.0–5.2
Mannose	5	16.14 ± 0.21	2.42 ± 0.14	65–70
Sorbitol	5	13.42 ± 0.31	2.50 ± 0.16	1.2–1.4
Crude glycerol	6.25^※^	24.78 ± 0.42	2.72 ± 0.10	0.25–0.3

^※^The concentration of crude glycerol was converted equally by the control group (glycerol, 5%).

As can be seen from Table 1, the yield of vitamin K_2_ is highest when glycerol is the carbon source. Sucrose can promote the growth of strains, likely because sucrose is better utilised by strains than other carbon sources. The results showed that although cells grew faster when sucrose was used as carbon source, vitamin K_2_ production was the highest when glycerol was used as the carbon source, which was consistent with the results of Tani *et al*.^24^ exploring the optimal medium for producing vitamin K_2_ by *Flavobacterium flavum*. Therefore, glycerol was chosen as the suitable carbon source for vitamin K_2_ production. However, the price of glycerol was high. In order to reduce the production cost, it was better to choose a cheaper carbon source. Therefore, crude glycerol was used to replace glycerol in this study. As shown in Table 1, crude glycerol and glycerol (t-test, data not presented) had the same beneficial effect on vitamin K_2_ production, which suggested that crude glycerol was an ideal alternative to glycerol. In addition, from an economic perspective, using crude glycerol instead of glycerol can reduce costs by 70%.

### Determination of crude glycerol concentration

**Figure 1:**
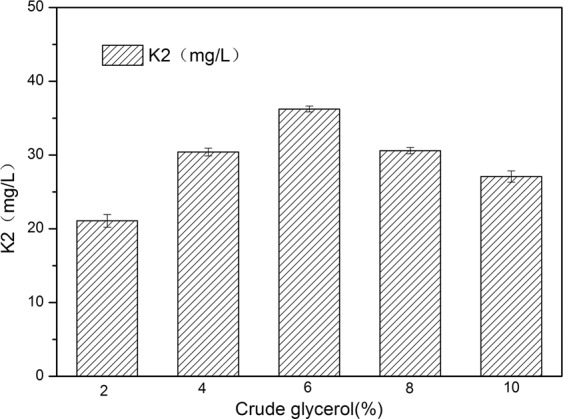
Effect of crude glycerol concentration on vitamin K_2_ production.

Using crude glycerol as a carbon source, different concentrations were selected: 2%, 4%, 6%, 8%, and 10%. Other culture conditions were unchanged. After shaking flask fermentation, vitamin K_2_ production was determined. The results (Fig. 1) showed that vitamin K_2_ yield was the highest when crude glycerol concentration was 6%.

### Effect of different nitrogen sources on vitamin K_2_ production

After the carbon source was determined, the nitrogen source was optimised. Nitrogen sources are essential nutrients for protein and nucleic acid synthesis, but different strains have different preferences for different nitrogen sources.

Table 2 shows that soybean peptone is the best nitrogen source for vitamin K_2_ synthesis. The prices of other nitrogen sources were cheap, but the yields of vitamin K_2_ were very low. Although the vitamin K_2_ yield of yeast extract as a nitrogen source was not as high as soybean peptone, yeast extract was beneficial to the growth of the strain. Nearly all relevant literatures had reported that soybean peptone was used with yeast extract to produce vitamin K_2_^10–12^. This study reconfirmed this conclusion. Yeast extract was rich in protein, amino acids, peptides, nucleotides, B vitamins, and trace elements. Its main function was to supplement nitrogen sources and provide various vitamins, amino acids, and growth factors for microbial growth. The nutritional varieties of soybean peptone was relatively few. The addition of yeast extract supplemented all types of growth factors needed for the growth of strains, increased the growth rate of the strains, and provided favourable conditions for the accumulation of vitamin K_2_. Based on the above results, not only peptone concentration but also yeast extract dosage should be investigated in subsequent studies.

**Table 2:** Effect of various nitrogen source on vitamin K_2_ production.

Carbon source	Concentration (%)	K_2_ yield(mg/L)	Biomass (g/L)	Prize(kg/dollar)
Soybean peptone	3	35.20 ± 0.61	2.80 ± 0.10	2.4–2.6
Yeast extract	3	32.53 ± 0.50	3.58 ± 0.11	3.0–3.5
Soybean peptone+ yeast extract	1.5 + 1.5	39.69 ± 0.52	3.80 ± 0.14	2.7–3.0
Beef extract	3	27.21 ± 0.42	2.62 ± 0.10	4.0–4.2
(NH_4_)_2_SO_4_	3	19.09 ± 0.21	2.22 ± 0.10	0.2–0.3
NH_4_Cl	3	24.21 ± 0.35	2.08 ± 0.11	0.12–0.14
NaNO_3_	3	22.11 ± 0.20	2.06 ± 0.10	0.5–0.6
NH_4_NO_3_	3	19.40 ± 0.26	2.42 ± 0.10	0.3–0.4
Soybean meal	3	18.32 ± 0.41	2.12 ± 0.11	0.5–0.6

### Determination of soybean peptone and yeast extract concentration

**Figure 2:**
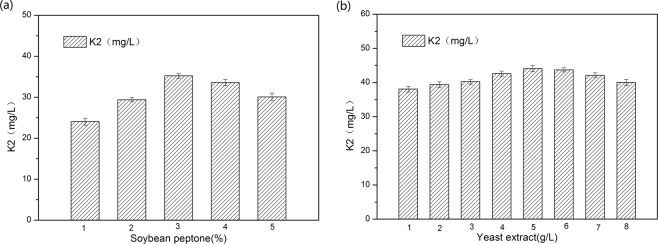
Effect of soybean peptone concentration and yeast extract concentration on vitamin K_2_ production. (**a**) soybean peptone; (**b**) yeast extract.

Because the nitrogen source has great influence on fermentation, and microbial growth and product synthesis have different requirements for nitrogen sources, the effect of nitrogen source concentration was further explored. As can be seen from Fig. 2(a), when the nitrogen source concentration is high, it can promote the synthesis of vitamin K_2_. However, excessive nitrogen sources inhibited the synthesis of vitamin K_2_, and 3% soybean extract was the best nitrogen source concentration for the synthesis of vitamin K_2_. Previous studies had found that when soybean peptone was added, yeast extract increased the production of vitamin K_2_. Therefore, the effect of yeast extract concentration on the synthesis of vitamin K_2_ was further investigated. As can be seen from Fig. 2(b), the optimal addition of yeast extract is 5 g/L.

### Box-Behnken design

On the basis of single factor test and BBD design principle, response surface analysis tests were designed at 17 test points to investigate crude glycerol (A), soybean peptone (B), and yeast extract (C). The experimental design and results are shown in Table 3. Variance for the quadratic design was analysed to check the validity of the model (Table 4).

**Table 3:** BBD experiments design matrix.

Code	A (%)	B (%)	C (g//L)	K_2_ (mg//L)
1	5	4	5	41.76 ± 0.23
2	6	3	5	44.15 ± 0.94
3	7	3	6	43.17 ± 0.43
4	6	2	4	41.48 ± 0.50
5	6	3	5	43.73 ± 0.60
6	5	3	4	42.65 ± 0.41
7	6	2	6	42.47 ± 0.67
8	6	4	6	41.77 ± 0.46
9	6	3	5	44.76 ± 0.47
10	5	3	6	41.30 ± 0.37
11	7	2	5	43.54 ± 0.64
12	7	3	4	41.48 ± 0.64
13	6	3	5	44.53 ± 0.63
14	6	3	5	44.10 ± 0.52
15	5	2	5	42.89 ± 0.83
16	6	4	4	43.03 ± 0.75
17	7	4	5	43.59 ± 0.75

**Table 4:** ANOVA of RSM.

Source	Sum of Squares	Mean Square	F Value	Probe (P) > F
Model	18.58	2.06	9.33	0.0038
A	1.26	1.26	5.71	0.0482
B	6.612E-003	6.612E-003	0.030	0.8677
C	6.125E-004	6.125E-004	2.767E-003	0.9595
AB	0.35	0.35	1.57	0.2501
AC	2.31	2.31	10.44	0.0144
BC	1.27	1.27	5.72	0.0481
A^2^	1.91	1.91	8.62	0.0218
B^2^	1.70	1.70	7.69	0.0276
C^2^	8.62	8.62	38.94	0.0004
Residual	1.55	0.22		
Lack of Fit	0.91	0.30	1.89	0.2727
Pure Error	0.64	0.16		
Cor Total	20.13			

The quadratic polynomial regression model equations of A, B, and C were obtained using Design-Expert software:

1 $$\begin{array}{ccc}{\rm{Y}} & = & 44.25+0.40\times {\rm{A}}-0.029\times {\rm{B}}+8.750{\rm{E}} \mbox{-} 003\times {\rm{C}}+0.30\times {\rm{A}}\times {\rm{B}}+0.76\times {\rm{A}}\times {\rm{C}}\\  &  & \,-0.56\times {\rm{B}}\times \,{\rm{C}}-0.67\times {{\rm{A}}}^{2}-0.64\times {{\rm{B}}}^{2}-1.43\times {{\rm{C}}}^{2}\end{array}$$

where Y was vitamin K_2_ yield, A was crude glycerol, B was soybean peptone, and C was yeast extract.

The results demonstrated that experimental values were distributed linearly with high correlation (R^2^ = 0.9730). Meanwhile, the results of variance analysis are shown in Table 4. They showed that the overall regression model established by the results was very significant (P < 0.01), and the lack of fit was not significant (P > 0.05), so the model was established. The results of Table 4 show that C^2^ reached very significant levels (P < 0.01), and A, AC, BC, B^2^, and A^2^ reached significant levels (P < 0.05). At the same time, it was inferred from F value that in the selected test range, the influence of three factors on the comprehensive score is A > B > C, and the interaction between factor A and B is the main interaction.

**Figure 3:**
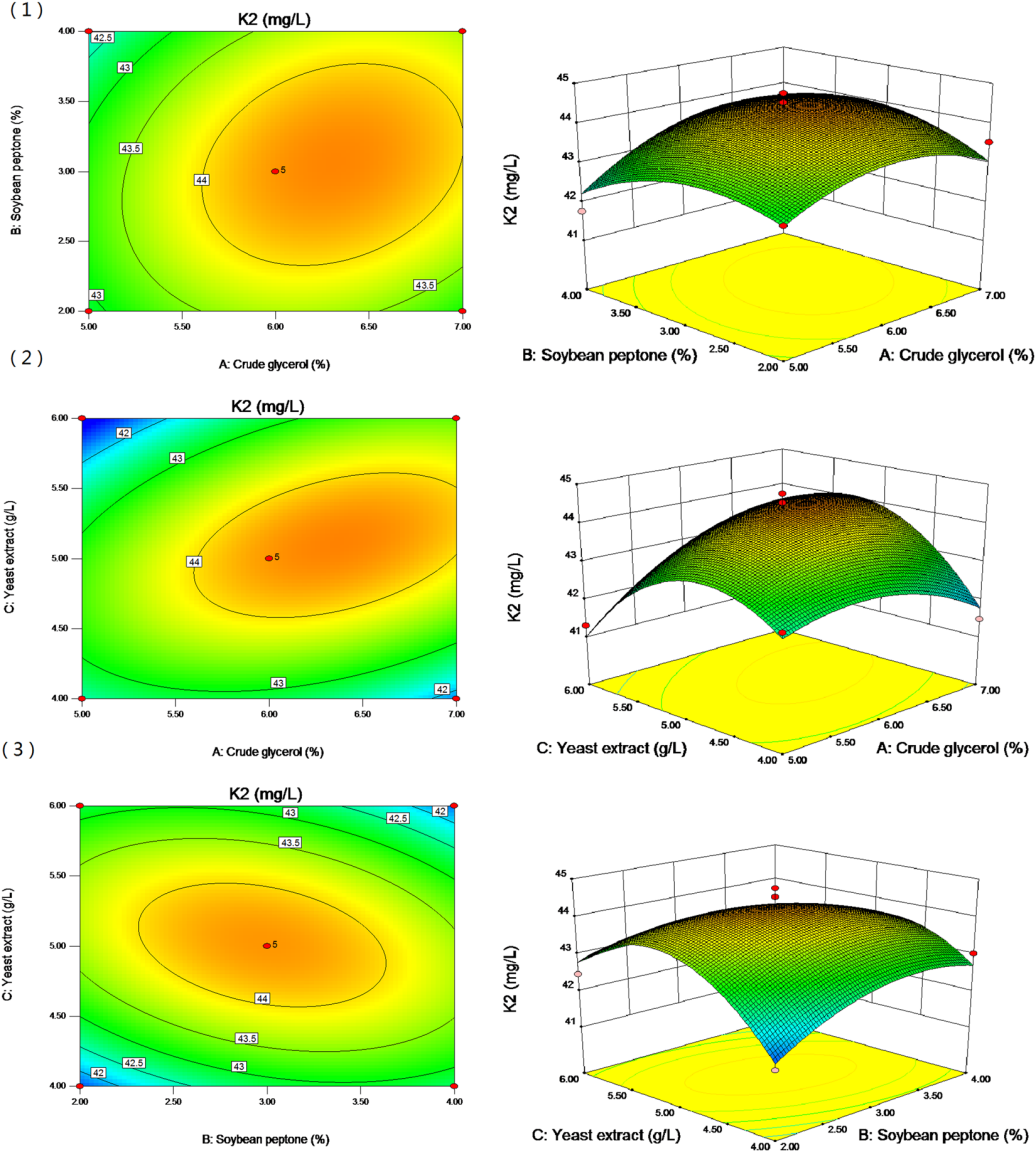
Surface and contour plots of mutual-influence. (**1**) effect of crude glycerol (A) and soybean peptone (B); (**2**) effect of crude glycerol (A) and yeast extract (C); and (**3**) effect of soybean peptone (B) and yeast extract (C).

According to the fitting model, the three-dimensional curves of different influencing factors were drawn (Fig. 3) to understand the interaction of various factors on vitamin K_2_ yield. The surface drawing demonstrates that the yield of vitamin K_2_ increases with the increase of crude glycerol concentration, but when the crude glycerol concentration increases past the optimal level, the yield of vitamin K_2_ decreases. With the increase of soybean peptone concentration and yeast extract concentration, the yield of vitamin K_2_ increases, but when the concentration of soybean peptone and yeast extract exceed the optimal amount, the yield of vitamin K_2_ decreases rather of increasing. According to the steepness of the surface drawing, the effect of crude glycerol concentration on vitamin K_2_ production was very significant, followed by peptone and yeast extract, which was consistent with the results of variance analysis.

The regression equation was solved by Design-Expert statistical software. The optimal medium was as follows: 6.35% crude glycerol, 3.02% soybean peptone, and 5.09 g/L yeast extract. The predicted value of vitamin K_2_ under this condition was 45.31 mg/L. For convenience, the crude glycerol concentration was set at 6.3%, the soybean peptone concentration was set at 3.0%, and the yeast extract concentration was set at 5.1 g/L. Under the optimal culture medium, the actual yield of vitamin K_2_ was 45.11 ± 0.62 mg/L, which was not significantly different from the theoretical predicted value (P > 0.05). Therefore, the results obtained by BBD were accurate and reliable, and had practical value.

### Time course of fermentation

**Figure 4:**
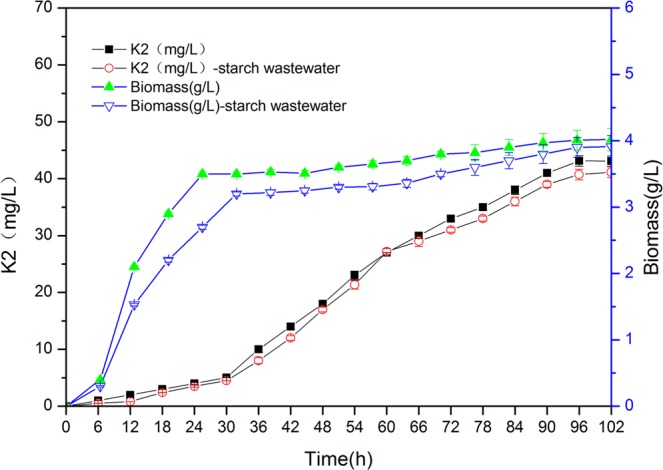
Kinetics of vitamin K_2_ production and biomass.

In order to further study the effect of crude glycerol on fermentation, *B. subtilis* Z-15 was fermented in a medium containing crude glycerol (6.3%) and a medium containing glycerol (5.04%), respectively. The biomass and vitamin K_2_ yield during fermentation were measured. The results are shown in Fig. 4. It can be seen that the accumulation of vitamin K_2_ is slow in the first 24 h and then enters a fast accumulation period in any medium. After 96 h, the accumulation of vitamin K_2_ reached maximum output. The growths of strains in different media are slow in the first 6 h, and then enter the fast growth period. After 30 h, the growths of strains enter the stable period. In conclusion, the growth and vitamin K_2_ synthesis of strains in both crude glycerol and glycerol media were similar. These results indicated that crude glycerol could replace glycerol well without affecting the growth or the synthesis of vitamin K_2_ of strains.

## Discussion

Crude glycerine is a by-product of alcohol fermentation and oil saponification. However, the high cost purification of crude glycerine is not economically feasible, which leads to a sharp drop in the price of crude glycerine—nearly becoming industrial waste. If not treated in time, it may become 17 new pollution sources, thus increasing the processing cost of biodiesel production enterprises and reducing economic benefits. This study proved that it was feasible to use crude glycerine as a substitute of pure glycerine to produce vitamin K_2_. This technology had the following advantages: (1) Using crude glycerine instead of pure glycerine in the production of K_2_ medium could save two thirds of the cost. (2) This technology may also offset the cost of crude glycerine treatment. (3) This technology could eliminate the pollution of crude glycerine in the environment. (4) With the continuous expansion of the scale of biodiesel, the output of crude glycerine also increases. However, there are not many ways to use crude glycerine. This technology could be used for reference in developing crude glycerine into other fermented products.

Crude glycerine from the biodiesel industry was used for producing vitamin K_2_ by *B. subtilis* Z-15. Glycerol was the most favourable carbon source for the synthesis of vitamin K_2_. Moreover, the use of crude glycerol did not adversely affect the synthesis of vitamin K_2_. Soybean peptone was the most favourable nitrogen source for the synthesis of vitamin K_2_. However, yeast extract was beneficial to the growth of the strain. The combination of soybean peptone and yeast extract was more conducive to the synthesis of vitamin K_2_. The optimal composition of the medium was obtained by RSM: 6.3% crude glycerol, 3.0% soybean peptone concentration, and 5.1 g/L yeast extract. Under the optimal culture medium, the vitamin K_2_ production was increased to 45.11 ± 0.62 mg/L. The fermentor test further proved that the use of crude glycerol neither affected the synthesis of vitamin K_2_ nor affected the growth of *B. subtilis*. In addition, from an economic point of view, using crude glycerol instead of glycerol can save 70% of the cost. These investigations may lay a foundation for reducing the pollution of crude glycerol, exploring a late model for vitamin K_2_ cleaner production.

## Materials and methods

### Strain

*B. subtilis* Z-15 (CICC 10260) was stored in our laboratory. The strain was maintained on slant medium at 5 °C.

### Industrial wastes

Crude glycerol was purchased from NanJing Changjiang Jiangyu Oil and Fat Co., Ltd, China. Its glycerol content was approximately 80%, and other impurities (water, partial polyglycerol, free alkali, organic matter, ash, etc.) were roughly 20%.

### Media

Slant medium, in g/L: glucose, 20; (NH_4_)_2_SO_4_, 10; NaCl, 5; and agar 18.

Seed medium (SM), in g/L: glucose, 30; peptone, 40; NaCl, 5; beef extract, 5; and yeast extract, 5.

Fermentation medium (FM), in g/L: glycerol, 50; soybean pepton, 30; yeast extract, 0.6; MgSO_4_•7H_2_O, 0.3; CaCl•H_2_O, 0.1; and K_2_HPO_4_, 0.3.

These media were sterilised by autoclaving at 121 °C for 20 min.

### Cultivation method

Three loopfuls of cells from the slant were inoculated into 500 mL conical flask containing 100 mL seed culture medium, sealed with gauze, then cultured in shaking bed at 37 °C and 120 r/min for 24 h. The cells in logarithmic growth phase were inoculated into fermentation medium, the inoculation amount was 5%, and they were cultured at 37 °C and 210 r/min for 96 h.

### Box-Behnken design

On the basis of single factor experiment, three factors which had significant influence on vitamin K_2_ yield were selected as independent variables: crude glycerol (A), soybean peptone (B), and yeast extract (C). The three factors were designed at three levels by Box-Behnken design (BBD) using Dseign-Expert 8.0.6 software, and the vitamin K_2_ yield (Y) was used as the response value. The regression coefficients of the equation were fitted by 17 groups of experiments.

### Time course of fermentation

Fermentation test was carried out in 5-L bioreactor (Baoxing Corp., Shanghai, China) containing 3 L fermentation medium. The flask was inoculated with 5% inoculum and cultured at 37 °C and 210 r/min for 96 h. Samples were taken every 6 h for measuring the yield of vitamin K_2_ and biomass.

### Analytical methods

The glycerol concentration in fermentation was determined by sodium periodate oxidation^24^.

The fermentation broth (10 mL) was transferred into a 250 mL conical flask with baffle. The mixture of isopropanol and n-hexane (1:2:4, v-v) was added to the fermentation broth. It was shaken in a shaking bed for 220 r/min for 30 min. After layering, the supernatant was rotated and steamed. The product was washed out with isopropanol and the volume was fixed in a 10 mL brown volumetric flask. Vitamin K_2_ content was analysed by UltiMate 3000 high performance liquid chromatography (HPLC). The working conditions of HPLC were as follows: chromatographic column: Brava-BDS C18 (250 mm × 4.6 mm, 5 micron); mobile phase: 100% methanol; flow rate: 1.0 mL/min; injection volume: 30 mL; column temperature: 50 °C; and detection wavelength: 270 nm.

Dry cell weight was determined after the cells were precipitated from 10 mL fermentation broth, washed once with distilled water, and dried at 105 °C overnight.
